# Excessive Weight Gain Followed by Catch-Down in Exclusively Breastfed Infants: An Exploratory Study

**DOI:** 10.3390/nu10091290

**Published:** 2018-09-12

**Authors:** Melanie W. Larsson, Mads V. Lind, Anni Larnkjær, Anette P. Due, Irina C. Blom, Jonathan Wells, Ching T. Lai, Christian Mølgaard, Donna T. Geddes, Kim F. Michaelsen

**Affiliations:** 1 Department of Nutrition, Exercise and Sports, University of Copenhagen, Rolighedsvej 26, 1958 Frederiksberg, Denmark; madslind@nexs.ku.dk (M.V.L.); ala@nexs.ku.dk (A.L.); icb@science.ku.dk (I.C.B.); cm@nexs.ku.dk (C.M.); kfm@nexs.ku.dk (K.F.M.); 2 Department of Nursing and Nutrition, University College Copenhagen, Sigurdsgade 26, 2200 N, Copenhagen, Denmark; adue@kp.dk; 3 UCL Institute of Child Health, University College London, 30 Guilford St, London WC1N 1EH, UK; jonathan.wells@ucl.ac.uk; 4 School of molecular Sciences, University of Western Australia, 35 Stirling Hwy, Crawley WA 6009, Australia; ching-tat.lai@uwa.edu.au (C.T.L.); donna.geddes@uwa.edu.au (D.T.G.)

**Keywords:** exclusively breastfed infants, weight-gain, infant growth, body composition, human milk composition, leptin, human milk intake, pediatrics, breastfeeding

## Abstract

Some infants experience excessive weight gain (EWG) during exclusive breastfeeding, but causes and consequences are unknown. The objective was to identify factors associated with early EWG. Infants with EWG (HW-group) were examined at 5, 9 and 18 mo and compared to a breastfed group with normal weight gain (NW-group). Anthropometry, body composition, milk and blood samples, and milk intake were measured. Mean body-mass-index-for-age *z*-scores (BAZ) increased 1.93 from birth to 5 mo in the HW-group (*n* = 13) while the NW-group (*n* = 17) was unchanged (−0.01). The HW-group had 70% more fat mass at 5 mo, and then showed marked catch-down in BAZ from 5 to 18 mo (−0.84). Milk intake at 5–6 mo did not differ between the groups. In the HW-group milk-leptin was lower at 5 mo and serum-leptin was considerably higher at 5 and 9 mo compared to the NW-group. Serum-leptin at 5 mo was positively associated with weight-for-age *z*-score (WAZ) and fat mass and negatively with WAZ change from 5 to 9 mo. In conclusion, breastfed infants with EWG had catch-down growth when other foods were introduced. Low milk-leptin in the HW-group may have stimulated appetite and milk intake when weight gain was high. High serum-leptin in the HW-group suggests early leptin resistance, which could impact cerebral regulation of energy intake. Larger studies are needed to confirm these results.

## 1. Introduction

Breastfed infants have a growth pattern different from formula fed infants. They grow more rapidly during the first 1–2 months (mo) and then more slowly—both weight gain and linear growth—in the first years [1,2,3]. There are also differences in body composition as breastfed infants accrue more fat than formula fed infants during the first 6 mo [4]. However, there is emerging evidence that human milk (HM) composition has an effect on growth, and some of the differences in growth within breastfed infants can be explained by differences in milk composition [5,6]. Recently there is emerging evidence that concentrations of the two hormones leptin and adiponectin in breast milk are related to appetite and infant growth [5,7].

Some infants experience excessive weight gain (EWG) during the period they are exclusively breastfed (EBF) and have a marked catch-down when complementary foods are introduced, suggesting that the EWG is caused by breastfeeding [8,9,10]. Studies investigating this group of infants are sparse and little is known about causes for the rapid weight gain and potential consequences. In the two case reports [8,9] it is suggested that a high protein intake is the cause. In an observational cohort study of 73 infants with EWG during EBF the growth pattern was different from the case reports because growth velocity continued to be high, also after complementary foods were introduced [11].

There is convincing evidence from several meta-analyses that a high weight gain during infancy, especially the first six months, is associated with an increased risk of overweight, obesity, and metabolic complications later in life [12,13,14,15]. However, most of these meta-analyses did not analyze the risk according to early feeding method. There is some evidence that breastfeeding reduces the risk of later overweight and obesity [16], but it is highlighted that observed associations could be due to residual confounding or reverse causation [17,18]. In a prospective population-based birth cohort study including 3000 children, overweight in infancy (≥1 standard deviation (SD) body-mass-index (BMI)-for-age (BAZ)) was associated with increased odds of childhood overweight regardless of feeding mode [19].

Although the case reports suggest that at least some breastfed infants with EWG show catch-down growth when other foods are introduced, it is important to examine if these infants are at risk of later obesity and metabolic complications. A small exploratory cohort of infants with EWG during EBF was established in order to identify predictors and consequences of the EWG and a cohort with normal weight gain was recruited for comparison. 

## 2. Materials and Methods 

### 2.1. Subjects and Recruitment

Participants were part of an ongoing prospective observational cohort study, the SKOT III cohort, consisting of two groups; a high weight gain group (HW-group) and a normal weight gain group (NW-group).

EBF infants considered to have excessively high weight-for-age were referred via Danish health workers who had been informed about the study through postal letters and social media from August 2015 to October 2016. High weight was defined as infants aged 4–6 mo having a weight-for-age *z*-score (WAZ) >2.00. The infants were qualified for enrollment to the HW-group if they had an increment of at least +1 SDS in WAZ within the first 5 mo postpartum. The NW-group was recruited through the National Civil Registry. Families from the Copenhagen area (*n* = 805) with infants born between 15 July 2016 and 31 August 2016 were invited by postal invitation to participate. Inclusion criteria for the NW-group were infants aged 4–6 mo with a WAZ between −1.0 and +1.0 SDS. Other inclusion criteria were similar for both groups: exclusively or fully breastfed (defined as receiving HM as their primary energy intake but were allowed to have water, vitamins, minerals and a maximum of one meal of formula per week) for at least 4 mo. At first visit the infants were to be 5–6½ mo old and have breastfeeding as the primary energy source not receiving more than two meals of solid food per day. Both HW- and NW-group mother-infant dyads were examined at infant’s age 5–6½ mo and 9 mo ± 2 weeks, and the HW-group further at 18 mo ± 4 weeks.

The study protocol was approved by the Regional Ethical Committee of the Capital Region of Denmark in accordance with the Helsinki declaration (H-15008948) and the Data Protection Agency (2015-57-0117 & 2015-57-0116) and written informed consent was obtained from parents.

### 2.2. Anthropometry

All anthropometric measurements at the three visits were carried out at the Department of Nutrition, Exercise and Sports, University of Copenhagen, Denmark. Weight and length from birth to 5 mo measured by health nurses and general practitioners, were reported by the parents and used for assessing recruitment criteria.

The weight of naked infants was measured using a digital pediatric scale giving an average of 40 measurements recorded to the nearest gram (Tanita BD 815 MA digital baby scale, Tanita Corporation, Tokyo, Japan). The following anthropometric measurements were performed in triplicate and the average was used: recumbent length to the nearest 0.1 cm (447 Infantronic Digital Infantometer, QuickMedical, Seattle, WA, USA), mid-upper-arm, head, lower leg, recumbent waist and thorax circumferences to the nearest millimeter (nonflexible Lasso-O tape measure, Child Growth Foundation, London, UK), and triceps and subscapular skinfold thickness to the nearest 0.1 mm (Harpenden skinfold caliper, Chasmors Ltd., London, UK). Sex and age specific *z*-scores; WAZ, length-for-age *z*-score (LAZ), BAZ, triceps skinfold-for-age (TSFZ) and subscapular skinfold-for-age (SSFZ) were obtained using the software WHO Anthro [20].

Mothers’ pre-pregnancy weight, pregnancy weight gain, and the fathers’ weight and height were self-reported, while the mothers’ weight and height were measured at all three visits (weight: Tanita digital medical scale, WB-100MA, Tanita Corporation, Tokyo, Japan; height: Static digital height measurer, 235 Heightronic Digital Stadiometer, Issaquah, WA, USA).

### 2.3. Body Composition

Body composition was measured using a tetrapolar Bioelectrical Impedance Analyzer (BIA) Quantum III (RJL Systems, Clinton Township, Detroit, MI, USA) which uses single frequency (50 kHz) between right hand and right foot to measure whole body resistance. Prediction of fat free mass (FFM) for each infant was completed using an equation developed by Lingwood et al. [21]. Details on BIA measurement and equation are included in Appendix A1; Supplemental method description.

### 2.4. Measurements of 24-h Infant Milk Intake

Mothers were instructed to use an electronic baby weighing scale (Tanita BD 815 MA, Tanita Corporation, Tokyo, Japan) for weighing their infants to measure the 24 h milk intake at 5 mo. For a period of 72 h mothers weighed their infants before and after each breastfeeding-session (each feed) without change in clothing or diaper. Calculation of intake in grams was done by subtracting weight of the infant before the feed from the weight after the feed. In cases where test weighing was not completed for all feeds, the intake was estimated using an average of intake per feed calculated from the mother’s registration. The first feed after the 72 h period was used as the end point for registration period, and in cases where these data were missing it was estimated from the three previous days. A corrected 24 h milk intake for each infant was determined by dividing the intervals in hours and multiplying by 24. No correction for infant insensible water loss was made, and therefore the milk intake is likely to be underestimated by 3–10% [22,23]. Feeding frequency and duration of each feed was derived from the test weighing report. Energy intake from foods and drinks other than HM was calculated using Dankost (Dankost version 3000, Dankost Ltd., Copenhagen, Denmark).

### 2.5. Human Milk Collection and Analysis

At the 5 and 9 mo visits the mothers were asked to collect 10 mL foremilk and 10 mL hind milk from either breast and on a second time point to complete expression of both breasts. Samples were collected in their homes in disposable bottles (2 × 80 mL/2 × 250 mL) using a manual breast pump (Type Harmony™, Medela AG, Baar, Switzerland) and stored at −20 °C. The samples were transported in a bag with an ice pack and stored at −80 °C.

HM analysis were carried out in one mixed sample of a full expression of right and left breast. Macronutrient and energy content was analyzed by mid-infrared spectroscopy using the HM analyzer (MIRIS HMA; Miris AB, Uppsala, Sweden). The analysis was performed according to manufacturer’s instructions. The milk was heated to 40 °C and homogenized by ultrasonic processing before injected into the system.

Biochemical analyses of milk leptin, adiponectin, lysozyme, sIgA and lactoferrin were carried out at the University of Western Australia. Milk adiponectin concentration was determined by Enzyme-Linked Immunosorbent Assay (ELISA) kits (RD191023100, BioVendor-Laboratorni medicina a.s., Brno, Czech Republic). Milk leptin (m-leptin) concentration was determined by the R&D systems leptin DuoSet ELISA kit (DY398, R&D Systems Inc., Minneapolis, MN, USA). The recovery of a known amount of leptin added to the milk samples was 97 ± 3.0% (*n* = 4). The detection limit of this assay was 0.016 ng/mL. Milk lactoferrin and secretory immunoglobulin A (sIgA) were measured with a sandwich ELISA method as previously described by Czank et al. [24]. Lysozyme concentration was measured with a modified turbidimetric assay [25]. Details on the methods are included in Appendix A2; Supplemental method description.

### 2.6. Blood Sampling and Analysis

Venous blood samples (10 mL) were taken at 5 and 9 mo visits. Local anesthetic patches (EMLA, AstraZeneca AB, Södertälje, Sweden) and instructions were given to the parents before first visit. If possible, infants were fasting 2 h before blood samples were taken and the time, amount and composition of last meal were recorded. Methods for blood analyses are described in Appendix A3; Supplemental method description. 

### 2.7. Feeding Patterns and Other Information

Information on parental characteristics and feeding practice was collected via interviews and questionnaires at 5 and 9 mo visits for both groups and at 18 mo visit for the HW-group. 

Appetite traits were measured at the 5 mo visit using the Baby Eating Behavior Questionnaire (BEBQ) [26]. BEBQ includes 4 appetite traits; food responsiveness (6 items), enjoyment of food (4 items), satiety responsiveness (3 items), slowness in eating (4 items) and general appetite (1 item). All items were scored on a 5-point Likert scale ranging from never (1) to always (5). High mean score signify a greater enjoyment of food, higher food responsiveness, slow eating, greater satiety responsiveness and larger general appetite. 

### 2.8. Statistical Analysis

Statistical analyses were carried out using STATATM12 (Stata Corporation, College Station, TX, USA). Significance was defined as *p*-value < 0.05. Parametric descriptive results are presented as means and SD, or mean and range. Independent *t*-test was used to test differences between the characteristics of the two study groups. Differences between groups for the anthropometric measures and blood parameters adjusted for sex (glucose, insulin, leptin, and Insulin-like Growth Factor-1 (IGF-I) and fasting time (glucose and insulin) were analyzed by general linear models. Non-normally distributed variables are presented as medians with interquartile range (IQR: 25th and 75th percentile), and Mann-Whitney U test used for comparing the study groups. Categorical variables are presented as number and percentage and comparisons were performed using Fisher’s exact test. Associations between the different macronutrients, between macronutrients/hormones and WAZ, BAZ and LAZ change, and between mothers BMI and milk composition were investigated by linear regression, for the groups separately and combined. 

## 3. Results

### 3.1. Subjects

In the HW-group 17 infants were referred according to the WAZ inclusion criteria. Four infants were excluded; two because they were long and had a BAZ < 2.00 and two because they had a relatively low weight gain from birth to 5 mo (both with birthweight > 4.00 kg and with a change in WAZ from birth to 5 mo of −0.16 and +0.6). Thus, the HW-group included 13 infants. Forty-two parents showed interest in participating in the NW-group and of these, 19 mother-infant dyads fulfilled the inclusion criteria. However, two infants were excluded; one had a birth weight of 2.67 kg (WAZ −1.3 SDS) followed by a catch-up with an increment in WAZ of 1.8 SDS and one had a birth weight of 4.66 kg (WAZ +2.5 SDS) followed by a catch down in WAZ of −2.9 SDS. Thus, the NW-group included 17 infants.

### 3.2. Anthropometry

Infants in the HW-group weighed on average 450 g more (*p =* 0.006) and were 1.3 cm longer (*p =* 0.045) at birth than the NW-group (Table 1) and BAZ was also higher (*p =* 0.025) (Table A1). 

**Figure 1:**
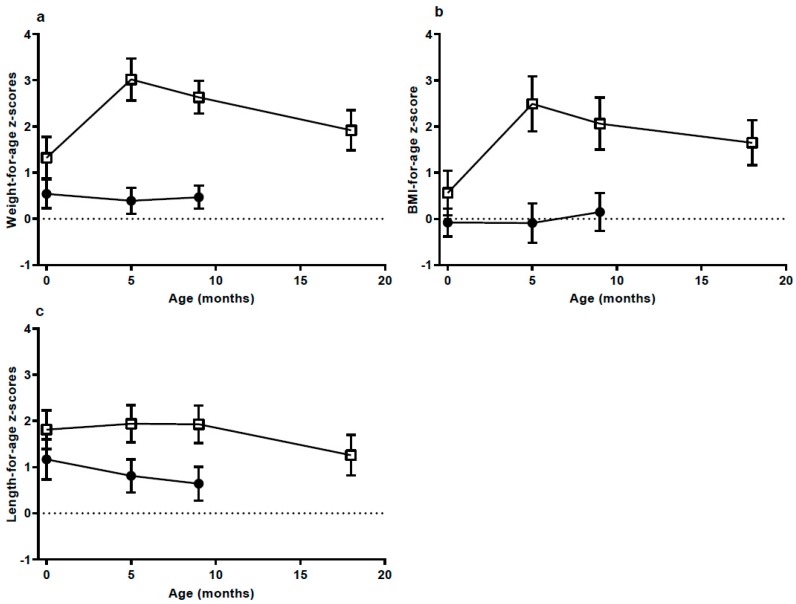
(**a**) Weight-for-age; (**b**) body mass index (BMI)-for-age and (**c**) length-for-age *z*-scores, according to groups presented as mean and 95% confidence interval at birth (High weight gain group (HW) *n* = 13; Normal weight gain group (NW) *n* = 17), 5 months (mo) (HW *n* = 13; NW *n* = 17), 9 mo (HW *n* = 13; NW *n* = 17) and 18 mo (HW *n* = 12 (weight and length for two infants were self-reported)). Data at 18 mo were not available for the NW group. HW = □ NW = ●.

From birth to the 5 mo visit weight gain was excessive in the HW-group (Figure 1). Mean weight gain was 6.6 kg and WAZ increased 1.71 units, reaching 3.02 at 5 mo. The three infants with the highest weight gain gained 7.6, 7.7 and 8.0 kg. The NW-group had an increase of 4.4 kg from birth to the 5 mo visit and their WAZ was unchanged (decreased 0.15 units; NS). From the 5 to 9 mo visit weight gain per week was the same in both groups (about 80 g/week, Table 1). WAZ and BAZ decreased from the 5 to 9 mo visit in the HW-group and continued to decrease from 9 to 18 mo, reaching mean values of 1.92 and 1.65, respectively (Figure 1 and Table A1). At 18 mo three infants in the HW-group still had a BAZ above +2.

### 3.3. Body Composition

**Figure 2:**
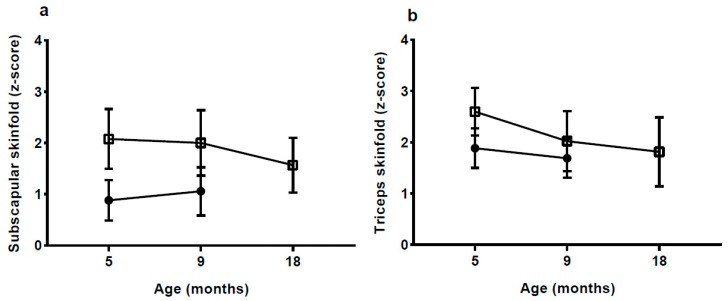
Subscapular and triceps skinfold *z*-scores according to groups, presented as mean and 95% confidence interval at 5 months (mo) (high weight gain group (HW) *n* = 13; normal weight gain group (NW) *n* = 17), 9 mo (HW *n* = 12; NW *n* = 17) and 18 mo (HW *n* = 9; NW *n* = 0). Data at 18 mo were not available for the NW group. HW = □ NW = ●.

Skinfold thickness and *z*-scores were significantly higher in the HW-group with the exception of triceps skinfold at 9 mo (Figure 2, Table 1 and Table A1).

**Figure 3:**
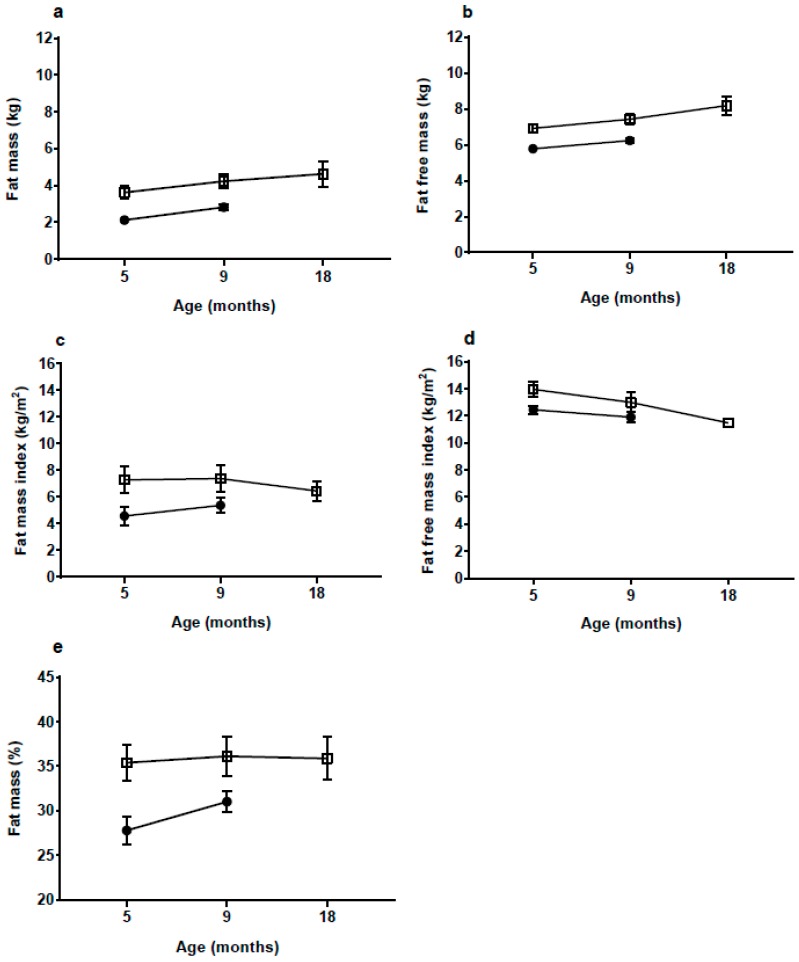
(**a**) fat mass; (**b**) fat free mass; (**c**) fat mass index; (**d**) fat free mass index and (**e**) fat mass percentage at 5 months (mo) (high weight gain group (HW) *n* = 12; normal weight gain group (NW) *n* = 17), 9 mo (HW *n* = 12; NW *n* = 15) and 18 mo (HW *n* = 7) according to group presented as mean and 95% confidence interval. Data at 18 mo were not available for the NW group. Bioimpedance values based on Lingwood equations [21]. HW = □ NW = ●

Fat mass (FM) and percentage FM (%FM), as measured by BIA, were significantly higher in the HW-group at both 5 and 9 mo (both *p <* 0.001, Figure 3, Table A1). In the HW-group %FM remained stable at approximately 35% from 5 to 18 mo, while in the NW-group it increased significantly (<0.001) from 5 to 9 mo. At 5 mo the HW-group had 1.49 kg or 70% more FM and 1.15 kg or 20% more FFM than the NW-group (Table A1). At 9 mo the HW-group had about 55% more FM than the NW-group.

### 3.4. Parental Characteristics

Mothers in the HW-group were significantly heavier pre-pregnancy and at 5 mo (Table 2). BMI was borderline significantly higher in mothers in the HW-group compared to mothers in the NW-group (*p =* 0.082). Two mothers were obese (BMI ≥ 30), both in the HW-group and four fathers were obese, two in each group.

### 3.5. Breastfeeding Duration, Milk Volume, Macronutrient and Hormone Content

The mean duration of exclusive or full breastfeeding for the HW- and NW-group was 5.14 mo (range: 3.69–6.46) and 5.54 mo (range: 3.46–6.46), respectively (*p =* 0.29). At the 9 mo visit, 83% of the HW- and 94% of the NW-group were still breastfed. At the 18 mo visit two infants in the HW group were still breastfed. The mean duration of any breastfeeding among the remaining ten mothers in the HW-group was 13.3 mo (range 8.2–18.0 mo).

Although the mean 24 h milk intake was 130 g (15%) higher in the HW-group the difference was not significant (*p =* 0.19, Table 3). There was no difference in HM intake per kg per day between the groups (*p =* 0.20). At the 5 mo visit, 88.5% of the infants received small amounts of complementary food and the median contribution to the total energy intake was not different in the HW-group compared to the NW-group (15.9% vs. 23.0%, *p =* 0.37). Milk intake data at 9 mo were incomplete and therefore not included.

There was no difference in milk fat, protein, lactose or energy content between the groups at 5 or 9 mo (Table 4). There were no associations between macronutrient content and 24 h milk intake (all *p >* 0.35) or frequency of breastfeeding at 5 mo (all *p >* 0.093, data not shown).

**Figure 4:**
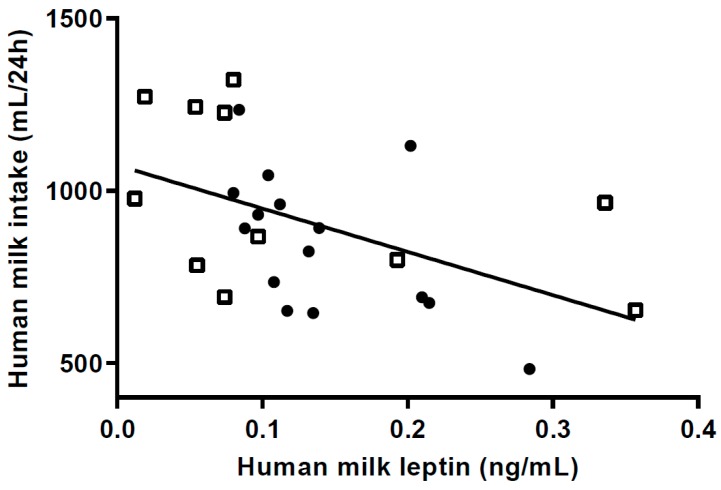
Scatterplot of 24 h milk intake in relation to milk leptin at 5 months (*p =* 0.011) (*n* = 11 in high weight gain group (HW); *n* = 15 in normal weight gain group (NW); HW = □ NW = ● Regression for HW group (*p =* 0.15); for NW group (*p =* 0. 0.031).

Median m-leptin content at 5 mo was significantly lower (~40%) in the HW-group (Table 4). At 9 mo, however, there was no difference in m-leptin content between the groups. M-leptin intake per kilo bodyweight at 5 mo was significantly lower in the HW-group (median (IQR) 8.53 ng/kg/day (4.30; 13.98) vs. 12.22 ng/kg/day (10.33; 17.76) respectively; *p =* 0.024). Twenty-four h milk intake (Figure 4) was negatively associated with m-leptin in the combined group at 5 mo (*p =* 0.01). Frequency of breastfeeding was not associated with either m-leptin intake (*p =* 0.23) or m-leptin concentration (*p =* 0.31) in the combined group.

Milk adiponectin content was not different between the two groups at 5 and 9 mo (Table 4) and there was no association with 24 h milk intake in the combined group (*p =* 0.86).

### 3.6. Maternal BMI and Milk Composition

In the combined group m-leptin content was higher in mothers with higher BMI at 5 mo (*p* < 0.001) and borderline higher at 9 mo (*p =* 0.057). For each increase in BMI unit leptin content increased 0.017 ng/mL at 5 mo. For mothers in the HW-group m-leptin at 5 mo was also positively associated with BMI (estimate [CI] 0.019 ng/mL [0.002; 0.036], *p =* 0.032, respectively), but not in the NW-group though the direction was the same (*p* ≥ 0.28). M-leptin at 5 mo was positively associated with maternal BMI (*p* < 0.001) and negatively with 24 h milk volume (*p =* 0.006) in a multiple regression analysis.

### 3.7. Associations between Milk Composition and Infant Growth

At 5 mo there were no significant associations between milk concentrations of macronutrients, energy and hormones and infant’s anthropometry (WAZ, BAZ or leight-for-age *z*-scores (LAZ)) or change in these *z*-scores from birth to the 5 mo visit (all *p >* 0.11, data not shown).

### 3.8. Breastfeeding Characteristics, Eating Behavior and Sleep Duration

The initiation of breastfeeding was without any problems for 85% (*n* = 11) and 88% (*n* = 15) of the mothers in the HW-group and NW-group, respectively. During the first 14 days 15% (*n* = 2) infants in the HW-group and 12% (*n* = 2) in the NW-group received small amounts of supplemental formula, but were EBF thereafter.

The HW-group was rated to enjoy food to a greater extent, having a higher overall appetite and being less sensitive to internal cues of satiety compared to the NW-group (Table 5).

At 5 mo maternal reported infant 24 h sleep duration was 1.7 h shorter in the HW-group compared to the NW-group (*n* = 16) (Median (IQR); 14.1 h (12.0; 15.0) vs. 15.8 h (14.5; 17.0) (*p =* 0.004). The number of night time awakenings were similar between groups (HW:median (IQR) 2 (1;3) vs. NW:3 (2;4), respectively; *p =* 0.10).

### 3.9. Infant Blood Samples

Infant serum leptin (s-leptin) was significantly higher, about double, in the HW-group compared to the NW-group at 5 mo (*p =* 0.004, Table 6). At 9 mo the difference remained significant (*p =* 0.006) though only 25% higher. The reduction in s-leptin from 5 to 9 mo in the HW-group was 62% compared to a 33% decrease in the NW-group (measured on individuals with measurements at both time points). There was no correlation between s-leptin and m-leptin at either 5 or 9 mo (both *p >* 0.62).

**Figure 5:**
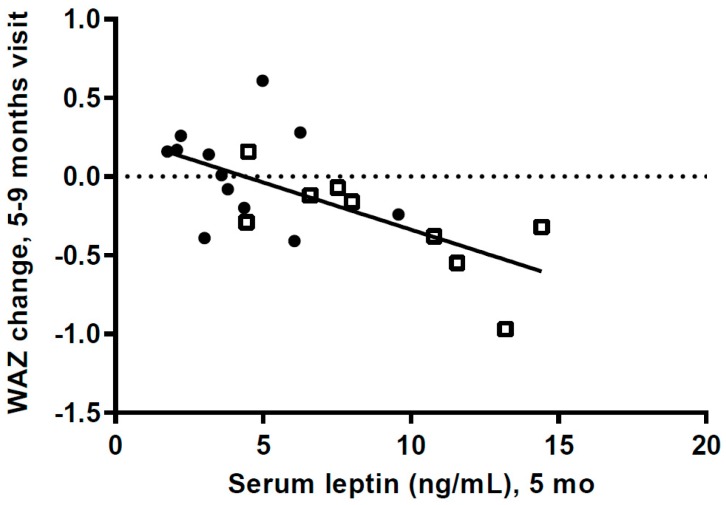
Scatterplot of change in weight-for-age *z*-scores from 5 months visit to 9 months visit versus serum leptin concentration at 5 months (*p =* 0.0015), (*n* = 9 in high weight gain group (HW); *n* = 12 in normal weight gain group (NW); WAZ: weight-for-age *z*-scores. HW = □ NW = ● Regression for HW group (*p =* 0.031); for NW group (*p =* 0.404).

At 5 mo s-leptin was positively associated with WAZ (*r* = 0.76, *p =* 0.0001) and FM (*r* = 0.68, *p =* 0.0007). At 9 mo only the association between s-leptin and WAZ was still significant (*r* = 0.49, *p =* 0.016). There were no associations between s-leptin and LAZ at either 5 or 9 mo (*p >* 0.31). S-leptin in the combined group at 5 mo was negatively associated with the change in WAZ from 5 to 9 mo (*p =* 0.0015), and the association was also significant for the HW-group alone (*p =* 0.03, Figure 5).

At 9 mo, albumin was higher (*p =* 0.018) and urea lower (*p =* 0.025) in the HW-group (Table A2). However, for the majority of the blood analysis there were no significant differences between the groups, either at 5 or 9 mo.

## 4. Discussion

We identified a group of breastfed infants who had a special growth pattern with EWG during the first 5 mo of EBF and catch-down during the following months, after beginning complementary foods. This growth pattern suggests that a key cause of the high weight gain should be found in factors related to HM intake and composition. There was surprisingly no difference in HM intake at the age of 5 mo between the groups. However, leptin content was significantly lower in the milk the HW-group received, while s-leptin was significantly higher in the HW-group compared to the NW-group. This suggests that leptin intake and metabolism could contribute in part to EWG in these infants.

The weight gain of infants in the HW-group was excessive. The mean weight gain per week was about 60% higher than in the NW-group, which had a weight gain close to the median weight gain in the WHO growth standard [20]. Catch-up growth is often defined as an increase in WAZ > 0.67 *z*-scores [15,27] and the mean increase in the HW-group from birth to the 5 mo visit was 2.5 times as high (1.71 *z*-scores). Interestingly, the two groups had almost the same gain in weight and length from the 5 mo to the 9 mo visit; a gain not very different from the median in the WHO growth standard velocities [20]. From the 9 to the 18 mo visit there was a continued decrease in WAZ and BAZ in the HW group, but to what degree this will continue and reach the normal range is not known. As outlined in the introduction, there is convincing evidence that early weight gain is associated with a higher risk of later overweight and obesity [15,27,28,29,30]. However, some studies show that breastfeeding has a modifying effect on the risk [31], while other studies do not [19]. The risk of later overweight and obesity in EBF infants with EWG is still unknown.

With a body FM of 3.6 kg at 5 mo, which is 70% more than the NW-group, the rate of fat deposition from birth to 5 mo must have been much greater in the HW-group. Although there was some decrease in BMI and skinfold *z*-scores from 5 to 18 mo in the HW group, %FM and fat mass index (FMI) continued to be high up to 18 mo, whereas the difference in fat-free mass index appeared to be decreasing. The medium-term effects of EWG therefore appear to manifest in both elevated length and adiposity. Links between accelerated linear growth and adiposity have been reported previously in the early post-natal period [32] and might indicate growth restriction early in pregnancy followed by the development of catch-up in late fetal life and infancy [33], resulting in ‘overshoot’ by early childhood of both length and adiposity.

The significant higher birth weight in the HW-group might also be a result of a different intra-uterine environment, partly because of the higher maternal pre-pregnancy weight in the HW-group. However, we find it unlikely that these differences between the groups could have a significant role in explaining the very large differences in post-natal growth between the groups.

HM intake is a strong driver of infant growth [34,35]. Therefore it was surprising, that milk intake was not significantly higher in the HW-group. The reason for this is likely to be the timing of measuring milk intake, at 5–6 mo, when infant growth had started to decline and complementary feeding had begun for most infants. However, though far from significant (*p =* 0.19), the intake was 15% higher in the HW-group. If there has been a difference of this magnitude throughout the first 5 mo it is likely to have an effect on growth. From 5 to 9 mo weight gain in the two groups was almost identical, and therefore the energy requirements might not have been different when milk intake was measured. Ideally, measurement of milk volume should have been carried out earlier e.g., between 1 and 4 mo of age when growth velocity was considerably higher; however, recruitment precluded this. Another reason that we did not find a significant difference could be the limited power in the study. In three of the cases reported earlier, 24 h milk intake at age 4 to 5.5 mo was high; 1123, 1132 and 1421 mL [9,10]. Mothers in the HW-group rated their infants as having higher appetite and being less sensitive to internal cues of satiety, which could reflect a higher milk intake earlier, but this is speculative.

Leptin influences the regulation of appetite by inducing satiety [36] providing a rationale for low m-leptin levels to be associated with increased milk intake in breastfed infants [7,36,37]. While transfer of m-leptin to infant plasma is one pathway by which leptin may regulate appetite control another is the presence of gastric leptin receptors which would more likely induce a more rapid infant response [7].

Lower levels of m-leptin content have been associated with greater infant length at 1 mo postpartum, and FM at 6 mo in EBF infants [38], although the association between milk leptin and growth is by no means conclusive [7]. In our study, M-leptin content in the HW-group was 38% lower than in the NW-group, and higher m-leptin was associated with lower milk intake at 5 mo, supporting the speculation that HW infants consumed more milk in the first 4 months post-partum.

Low m-leptin levels may lead to higher milk intakes as a result of greater suckling pressure (frequency) in the HW-gain group. This phenomenon is generally associated with maternal malnutrition and infant catch-up growth [39,40] reducing milk leptin content so as to lower infant satiety and promote higher milk transfer. However increased sucking frequency has also been shown in the 3 month old infants of obese mothers [41]. Moreover, appetite has a strong genetic basis [41], which could account for correlations between maternal BMI and infant appetite. These factors could explain the substantial catch-up overshoot if the mother is in fact well-nourished and able to provide more milk [42,43]. Further these combined findings support the assumption that the EWG and increased accrual of fat in our HW-group could be in part driven by a high intake of HM during the months where there was rapid weight gain, despite our failure to show differences in milk intake. While we found a positive association of maternal BMI with m-leptin, as reported by others [44], this included all of the participants. Comparing between groups, the HW-group mothers had significantly lower m-leptin than the NW-group, despite having higher average BMI. This again suggests some facultative response in milk composition in the HW-group, potentially mediated by elevated infant suckling and/or genetic factors.

Mean infant s-leptin values were markedly higher in the HW-group at both 5 and 9 mo. This can be explained at least partly by the higher FM in the HW-group, as s-leptin is mainly produced by fat tissue [36,37]. Within the whole sample, we also observed strong correlations between 5 mo s-leptin values and infant weight and FM. This is consistent with other studies reporting associations between s-leptin and BMI and %FM in early childhood [45,46,47].

A small group of infants have been shown to have very high leptin levels during the first 6 mo, with a value for the 90th percentile about four times as high as the median (11.86 vs. 2.81 ng/mL) [48]. Our values show comparable differences between the HW- and the NW-group (7.77 vs. 3.70 ng/mL). Recently, three single nucleotide polymorphisms (SNPs) have been shown to influence s-leptin levels in infants below 6 mo [49]. Thus, we speculate that the very high s-leptin levels in our HW-group might have a genetic basis [49]; however, this needs to be confirmed.

Leptin is considered an anorexigenic hormone but it has been suggested that during early infancy some infants are likely to develop leptin resistance. It has been suggested, based on studies with mice, that high s-leptin levels, during an early postnatal leptin surge, can influence the hypothalamus and thus increase appetite and thereby growth [50,51,52]. The very high s-leptin values in some 0–6 mo old infants [48], and the very high values in our HW-group could reflect that some infants have a postnatal leptin surge. However, this is speculative.

We do not know the s-leptin levels of the HW infants when weight gain was very high during the first postnatal months. However, if leptin levels were very high and leptin resistance had developed it could have an effect on milk intake and growth. The high s-leptin levels at 5 mo could also be a result of the high FM. Our data, showing a highly significant negative association between s-leptin levels at 5 mo and weight gain from 5 to 9 mo, could be a result of decreased leptin resistance when other foods were introduced, with s-leptin then having an anorexigenic effect.

Overall, it is remarkable that in this relatively small cohort there were significant differences in both m-leptin and s-leptin levels, suggesting that leptin could have a role in the excessive weight gain in the HW group. However, the potential mechanism we have discussed here are speculative and there is a need for larger studies to investigate the potential role of leptin in excessive weight gain in exclusively breastfed infants.

Other potential factors that could have an effect on the high weight gain in the HW-group are the effect of milk composition on the microbiota. Both HM oligosaccharides (HMO) and leptin have been shown to be independently associated with breastfed infants gut bacteria, which could have an effect on intestinal energy harvest [5,53,54]. It has been shown that HMO can influence weight gain and body fat accretion, most likely through an effect on the microbiota [55]. Differences in early energy metabolism and expenditure between the groups could also influence weight gain. Genes or epigenetics might also play a role, e.g., some SNPs have been shown to influence s-leptin levels [49].

A limitation is that it was not possible to identify, recruit, and arrange clinical visits before the age of 5 to 6 mo, by which time growth velocity was decreasing and some infants had started on complementary foods. If we had been able to measure milk intake when growth velocity was considerably higher, assessment of milk intake, milk composition and blood would most likely have given a better understanding of why these infants experience such a high weight gain. Furthermore, the power of the study was not very high due to the relatively small number of infants included. We have made multiple comparisons, but as the number of infants in the groups was small and as we have had an explorative approach, we have not controlled for this.

Aside from these limitations, this study is the first to bring valuable knowledge for future studies designed to investigate the causes of EWG in exclusively breastfed infants further. A strength of the study is, that it is the first cohort of breastfed infants with EWG during the first 6 mo followed by catch-down, which has been followed longitudinally with detailed measurements of growth, body composition, HM intake and blood samples.

## 5. Conclusions

In conclusion, our exploratory study suggests that leptin could influence the early EWG and later catch-down in the HW-group but the mechanisms are not known. There is a need to explore this in future studies, especially to understand how leptin is influencing early postnatal growth. The very high weight gain in the HW-group might increase the risk of later overweight and obesity and metabolic complications. The mean BAZ value was within the higher normal range at 18 mo, but three infants still had a BAZ above +2. Thus, it seems like at least some of the infants with a very high infant weight gain reach a weight within the normal range within the following years. In that perspective, it is interesting that in a large study, measuring BMI at 8 to 10 years, there was a highly significant inverse association between s-leptin values in infancy and childhood BMI suggesting a programming effect [56]. Another important finding is that we observed no differences between the groups in many metabolic parameters, including blood lipids, liver parameters and inflammation markers at 5 or 9 mo, suggesting that there were no major metabolic disturbances in the HW-group. However, long-term follow up is needed to assess the long-term risk of overweight and obesity in these infants.

## Figures and Tables

**Table 1:** Infant anthropometry according to the high weight gain and normal weight gain group ^1^.

	HW Group	NW Group	*p*-Value ^2^
At birth			
*n*	13	17	
Gender (girls/boys) *n* (%)	5 (38.5)/8 (61.5)	10 (58.8)/7 (41.2)	0.27
Gestational age, weeks	40.7 [40.5, 41.1]	40.4 [39.7, 41.3]	0.42
Cesarean delivery, *n* (%)	4 (33.3)	2 (11.8)	0.20
Weight, kg	3.99 ± 0.41	3.55 ± 0.31	**0.006**
Length, cm	53.0 ± 1.4	51.7 ± 1.7	**0.045**
5 months visit			
*n*	13	17	
Age, months	5.6 ± 0.5	5.9 ± 0.3	0.054
Weight, kg	10.60 ± 0.88	7.89 ± 0.50	**<0.001**
Length, cm	70.5 ± 2.2	68.1 ± 2.1	**0.009**
Weight velocity birth to 5 months visit, g/week	271.9 ± 32.4	169.2 ± 17.1	**<0.001**
Skinfold thickness, triceps, mm	15.1 ± 2.0	13.2 ± 1.8	**0.011**
Skinfold thickness, subscapular, mm	10.9 ± 2.1	8.6 ± 1.4	**0.001**
9 months visit			
*n*	12	17	
Age, months	9.0 ± 0.3	9.1 ± 0.2	0.48
Weight, kg	11.65 ± 0.79	9.03 ± 0.37	**<0.001**
Length, cm	75.7 ± 1.9	72.5 ± 2.0	**<0.001**
Weight velocity 5 to 9 months visit, g/week	80.4 ± 22.47	81.9 ± 20.76	0.93
Skinfold, triceps, mm	12.9 ± 2.5	12.2 ± 1.9	0.26
Skinfold, subscapular, mm	10.2 ± 2.2	8.5 ± 1.5	**0.019**
18 months visit			
*n*	10/12 ^3^		
Age, months	18.2 ± 0.7		
Weight, kg	13.41 ± 1.36		
Length, cm	85.3 ± 2.4		
Weight velocity 9 to 18 months visit, g/week	43.3 ± 23.9		
Skinfold, triceps, mm	11.4 ± 2.1		
Skinfold, subscapular, mm	8.5 ± 1.2		

^1^ Values are expressed as mean ± standard deviation, median [25th; 75th percentile] or number (percentage) as appropriate. HW: high weight gain group; NW: normal weight gain group. ^2^ *p*-values for differences between groups analyzed by Fisher’s exact Chi-squared test for proportions, by independent *t* test for age at visit, and by general linear model adjusted for sex for all other variables. ^3^ For two infants weight and length at 18 mo were self-reported and there were no other anthropometric data for these two infants.

**Table 2:** Parental characteristics according to the high weight gain and normal weight gain group ^1^.

	HW Group	NW Group	*p*-Value ^2^
	(*n* = 13)	(*n* = 17)	
*Maternal* ^3^			
Age at birth, years	32.5 ± 3.9	33.7 ± 3.2	0.40
Parity, >1 (%)	53.9	52.9	0.96
Pre-pregnancy weight, kg	71.9 ± 12.0	60.6 ± 6.0	**0.002**
Height, cm	170.2 ± 5.5	165.8 ± 7.3	0.077
Pre-pregnancy BMI, kg/m^2^	24.2 [22.1, 26.6]	22.2 [21.2, 22.5]	0.082
BMI ^4^ < 18.5, *n* (%)	0 (0)	1 (5.9)	0.24
BMI 18.5–24.99, *n* (%)	9 (69.2)	15 (88.2)	
BMI 25–29.99, *n* (%)	2 (15.4)	1 (5.9)	
BMI ≥ 30, *n* (%)	2 (15.4)	0 (0)	
Gestational weight gain, kg	14 [12, 17]	15 [13, 18]	0.40
Weight, kg at birth—estimated	86.9 ± 12.6	76.4 ± 6.2	**0.014**
Weight, kg at 5 months	75.7 ± 16.8	62.2 ± 5.5	**0.004**
Weight, kg at 9 months	70.9 ± 15.9	61.9 ± 5.9	0.053
Weight, kg at 18 months	76.9 ± 15.7		
*Paternal*			
Age at birth, years	35.6 ± 5.3	34.9 ± 5.9	0.75
Weight, kg	88.8 ± 12.0	87.4 ± 30.6	0.88
Height, cm	182.3 ± 7.8	182.2 ± 7.3	0.98
BMI, kg/m^2^	26.7 (24.6, 29.3)	23.2 (22.2, 26.9)	0.088

^1^ Values are expressed as mean ± standard deviation, median [25th; 75th percentile] or number (percentage) as appropriate. HW: high weight gain group; NW: normal weight gain group. ^2^ *p*-values for differences between groups by independent *t* test or Mann-Whitney U test as appropriate. Fisher’s exact Chi-squared test was used for proportions. ^3^ Pre-pregnancy weight and gestational weight loss are self-reported. ^4^ Body mass index (BMI).

**Table 3:** Intake of human milk and complementary food at 5–6 months, in the high weight gain and normal weight gain group ^1^.

	HW Group	NW Group	*p*-Value ^2^
	11	15	
*24-h test weighing*			
Age, 24-h test weighing, months	5.5 ± 0.4	6.1 ± 0.4	**0.003**
24-h milk intake, g	981.9 (653.8–1321.4)	852.0 (482.7–1234.7)	0.19
Milk 24-h intake, g/kg/day	94.4 (58.0–124.3)	107.6 (64.2–149.0)	0.20
Average milk intake per feed, g	118.3 (58.8–208.3)	111.8 (51.2–201.5)	0.71
Breastfeeding frequency, *n* per day	8.9 (6.0–12.0)	8.1 (5.0–11.6)	0.52
Duration of breastfeeding meal, min	10.9 (3.0–22.4)	9.2 (3.7–19.7)	0.36
*Energy intake from human milk and complementary food*			
Energy intake from human milk, kcal/day	618.6 (323.6–962.5)	584.9 (330.6–981.6)	0.66
Exclusively breastfed at test weighing, *n* (%)	3 (27.3)	0 (0)	0.063
Energy intake from complementary foods, kcal/day ^3^	131.2 (40.7–263.1)	255.7 (7.9–825.0)	0.30
Total energy intake from human milk and complementary feeding, kcal/day ^3^	703.9 (424.0–1176.8)	840.6 (551.5–1222.0)	0.19

^1^ Values are expressed as mean ± standard deviation or mean (range) as appropriate. HW: high weight gain group; NW: normal weight gain group. ^2^ Comparing groups by independent *t* test and Mann-Whitney U test as appropriate. ^3^ HW *n* = 8, NW *n* = 15.

**Table 4:** Macronutrient, hormone and protein content of human milk at 5 and 9 months, in the high weight gain and normal weight gain group ^1^.

	HW Group	NW Group	*p*-Value
*5 months milk sampling*			
*n*	11	17	
Age, months	5.8 ± 0.7	5.8 ± 0.4	0.92
*9 months milk sampling*			
*n*	8	14	
Age, months	8.7 ± 0.7	9.2 ± 1.0	0.26
*Macronutrient content and energy*			
Fat, g/L			
5 mo	30.2 ± 15.9	36.1 ± 8.7	0.28
9 mo	43.7 ± 15.8	33.2 ± 8.9	0.13
Protein, g/L			
5 mo	7.3 ± 1.1	8.0 ± 1.1	0.15
9 mo	8.0 ± 1.0	8.6 ± 1.7	0.31
Lactose, g/L			
5 mo	76.3 ± 2.2	75.8 ± 1.9	0.55
9 mo	74.5 ± 1.9	73.8 ± 4.6	0.64
Energy, kilocal/L			
5 mo	625.5 ± 142.2	680.3 ± 78.2	0.26
9 mo	746.4 ± 140.7	649.6 ± 86.6	0.13
*Hormones and proteins* ^2^			
Adiponectin, ng/mL			
5 mo	14.65 [13.03, 15.56]	13.23 [10.54, 14.65]	0.36
9 mo	17.52 [15.88, 20.80]	20.80 [11.92, 29.23]	0.76
Leptin ng/mL ^2^			
5 mo	0.07 [0.05, 0.15]	0.12 [0.10, 0.14]	**0.045**
9 mo	0.10 [0.06, 0.17]	0.12 [0.09, 0.19]	0.34
Lysozyme g/L ^2^			
5 mo	0.06 ± 0.01	0.08 ± 0.06	0.24
9 mo	0.07 ± 0.02	0.01 ± 0.02	0.076
sIgA g/L			
5 mo	0.32 ± 0.11	0.30 ± 0.10	0.61
9 mo	0.35 ± 0.13	0.42 ± 0.15	0.31
Lactoferrin g/L			
5 mo	2.31 ± 1.30	2.54 ± 1.23	0.64
9 mo	3.89 ± 1.63	4.71 ± 2.69	0.44

^1^ Values are expressed as mean ± standard deviation or median [25th; 75th percentile] as Appropriate. HW: high weight gain group; NW: normal weight gain group. ^2^ Comparing groups by independent *t* test and Mann-Whitney U test as appropriate.

**Table 5:** Baby Eating Behaviour Questionaire at 5 months ^1^.

Baby Eating Behaviour Questionaire Scale	HW Group	NW Group	*p*-Value ^2^
*n*	13	17	
Food responsiveness	1.83 [1.17, 2.50]	1.50 [1.33, 1.83]	0.47
Enjoyment of food	5.00 [4.75, 5.00]	4.50 [4.00, 4.75]	**0.005**
Satiety responsiveness	2.33 [2.00, 2.33]	2.67 [2.33, 3.00]	**0.010**
Slowness in eating	1.50 [1.25, 2.25]	2.00 [1.25, 2.50]	0.78
General appetite	5.00 [4.00, 5.00]	4.00 [3.00, 4.00]	**0.007**

^1^ Data are presented as median [25th; 75th percentile]. HW: high weight gain group; NW: normal weight gain group. ^2^ Comparing groups by independent Mann-Whitney U test.

**Table 6:** Blood analysis at 5 and 9 months according to high weight gain and normal weight gain group ^1^.

	HW Group	*n* (8–11)	NW Group	*n* (12–14)	*p*-Value ^2^
Glucose, mmol/L					
5 mo	6.17 (0.65)	11	5.82 (0.57)	13	0.35 ^3^
9 mo	5.59 (0.44)	11	5.72 (0.78)	14	0.87 ^3^
Insulin, pmol/L					
5 mo	50.9 [25.3,88.8]	10	39.7 [11.1, 49.0]	12	0.099 ^3^
9 mo	23.2 [6.7, 43.3]	9	38.8 [24.0, 68.2]	13	0.41 ^3^
Ghrelin, pg/mL					
5 mo	1.66 (0.638)	10	2.06 (0.440)	11	0.11
9 mo	1.95 (0.495)	9	1.94 (0.473)	13	0.96
Leptin, ng/mL					
5 mo	7.77 [4.94, 11.6]	10	3.70 [2.62, 5.52]	12	**0.004** ^3^
9 mo	2.99 [2.09, 4.18]	10	2.36 [1.43, 2.98]	14	**0.006** ^3^
Adiponectin, ng/mL					
5 mo	15086 [12,089, 19361]	10	21646 [14,850, 26,091]	13	0.083
9 mo	9847 [7616, 11,964]	10	12760 [9723, 18,649]	14	0.16
IgF-BP3, µg/mL					
5 mo	2.215 (0.423)	10	1.993 (0.433)	12	0.24
9 mo	2.327 (0.407)	9	2.312 (0.661)	13	0.95
IgF-1, ng/mL					
5 mo	29.2 [22.0, 40.4]	10	23.9 [22.4, 36.5]	12	0.50 ^3^
9 mo	31.0 [26.7, 32.3]	9	37.4 [29.1, 40.7]	13	0.44 ^3^
C-peptid, pmol/L					
5 mo	282 [217, 453]	10	311 [170, 373]	12	0.47
9 mo	215 [178, 309]	9	361 [201, 563]	13	0.22

^1^ Values are expressed as mean ± standard deviation or median [25th; 75th percentile] as appropriate. HW: High weight gain group; NW: normal weight gain group. ^2^ *p*-values for differences between groups by independent *t* test and Mann-Whitney U test as appropriate. ^3^ *p*-values for differences between groups adjusted for sex and fasting time (glucose and insulin) analyzed by general linear models.

## Appendix A. Supplemental Methods Description

### A.1. BIA Measurements

The infant was placed on an examination couch without wearing metal or any persons touching the skin of the infant. The signal electrodes were placed on the right hand behind the knuckle of the middle finger and on the right foot behind the toe next to the big toe. The detecting electrodes were placed on the right wrist next to the ulna head and on the right ankle between the medial and lateral malleoli. The bioimpedance measurements were then performed.

Details on Lingwood equation:

(W = weight in kg, S = sex, L = length, R_50_ = impedance variable)

$$\;\mathrm{FFM}\;=\;2.203\;+\;0.334W\;–\;0.361S\;+\;\frac{0.185L^{2}}{R_{50}}\;$$

Fat mass (FM) was calculated as weight − FFM and %FM = 100(W − FFM)/W. Fat free mass index (FFMI) and fat mass index (FMI) was calculated using the following equations:

$$\;\mathrm{FFMI}\;=\;\frac{fat\;free\;mass}{height^{2}}\;)\;\mathrm{FMI}\;=\;(\frac{\mathrm{kg}}{m^{2}}\frac{fat\;mass}{height^{2}}\;\left(\frac{\mathrm{kg}}{m^{2}}\right)$$

### A.2. Milk Analyses

Skim milk samples were diluted 10-fold with 0.1 M of Na2 HPO4/1.1 mM of citric acid (pH 5.8) buffer. Twenty-five microliters of standards or diluted skim milk samples were placed into the wells of a plate (Greiner Bio-One, Frickenhausen, Germany). A 175 μL of Micrococcus lysodeiltikus suspension (0.075% *w*/*v*, ATCC No. 4698, Sigma, St. Louis, MA, USA) was added into each well and plate was incubated on a shaker at RT for 1 h. The absorbance was measured at 450 nm.

### A.3. Blood Analyses

For the glucose, hemoglobin, leptin, adiponectin and total ghrelin analysis blood samples were taken in EDTA tube. Inhibitors (Pefabloc, DDP-IV and Trasylol; Sigma-Aldrich, Gentofte, Denmark) were added for the ghrelin analysis. Tubes for analyzing ghrelin, adiponectin and leptin were put on ice immediately after blood draw and kept on ice until plasma separation. Whole blood glucose and hemoglobin concentration were analyzed immediately after blood sampling using Glucose 201 analyzer (Hemocue Denmark, Brønshøj, Denmark) and automated hematology analyzer (Sysmex KX-21NTM, Kobe, Japan), respectively. All other blood samples were stored at −80 °C for later analysis. ELISA was used to measure plasma leptin, adiponectin (R&D Systems, Inc., Minneapolis, MN, USA), and total ghrelin (EMD Millipore Corporation, Millipore, MO, USA).

## Appendix B. Supplemental Tables

**Table A1:** Anthropometric values according to high weight-gain and normal weight-gain group and age ^1^.

	HW Group	NW Group	*p*-Value ^2^
*At birth*			
*n*	13	17	
Weight-for-age *z*-score	1.32 ± 0.75	0.54 ± 0.61	**0.006**
BMI-for-age *z*-score	0.56 ± 0.80	−0.08 ± 0.59	**0.025**
Length-for-age *z*-score	1.81 ± 0.70	1.17 ± 0.85	**0.037**
*At 5 months*			
*n*	13	17	
Weight-for-age *z*-score	3.02 ± 0.76	0.39 ± 0.55	**<0.001**
BMI-for-age *z*-score	2.49 ± 0.99	−0.09 ± 0.83	**<0.001**
Length-for-age *z*-score	1.94 ± 0.67	0.81 ± 0.70	**<0.001**
Waist, cm	50.3 ± 2.2	42.5 ± 1.8	**<0.001**
Mid upper arm circumference, cm	17.7 ± 0.9	14.8 ± 0.9	**<0.001**
Head circumference, cm	45.6 ± 1.5	43.7 ± 1.2	**0.001**
Lower leg circumference, cm	22.2 ± 1.6	18.1 ± 1.1	**<0.001**
Thorax circumference, cm	49.5 ± 1.9	43.5 ± 1.4	**<0.001**
Skinfold, triceps *z*-score	2.60 ± 0.77	1.89 ± 0.75	**0.018**
Skinfold, subscapular *z*-score	2.08 ± 0.96	0.88 ± 0.77	**0.001**
Bioimpedance, *n*	12	17	
Fat free mass, kg	6.92 ± 0.39	5.78 ± 0.25	**<0.001**
Fat mass, kg	3.61 ± 0.54	2.12 ± 0.33	**<0.001**
Fat free mass index	13.96 ± 0.93	12.46 ± 0.57	**<0.001**
Fat mass index	7.27 ± 1.00	4.56 ± 0.71	**<0.001**
Fat mass percentage, %	35.40 ± 3.18	27.78 ± 3.14	**<0.001**
*At 9 months*			
*n*	12	17	
Weight-for-age *z*-score	2.63 ± 0.56	0.47 ± 0.49	**<0.001**
BMI-for-age *z*-score	2.06 ± 0.89	0.15 ± 0.80	**<0.001**
Length-for-age *z*-score	1.93 ± 0.64	0.64 ± 0.71	**<0.001**
Waist, cm	50.0 ± 1.9	43.6 ± 1.7	**<0.001**
Mid upper arm circumference, cm	17.6 ± 1.2	15.0 ± 0.7	**<0.001**
Head circumference, cm	47.7 ± 1.3	45.3 ± 1.4	**<0.001**
Lower leg circumference, cm	21.3 ± 1.3	18.1 ± 0.9	**<0.001**
Thorax circumference, cm	49.8 ± 1.9	45.2 ± 1.1	**<0.001**
Skinfold, triceps *z*-score	2.0 ± 0.9	1.7 ± 0.8	0.39
Skinfold, subscapular *z*-score	2.00 ± 1.01	1.09 ± 0.93	**0.021**
Bioimpedance, *n*	12	15	
Fat free mass, kg	7.43 ± 0.47	6.25 ± 0.21	**<0.001**
Fat mass, kg	4.22 ± 0.61	2.82 ± 0.28	**<0.001**
Fat free mass index	13.01 ± 1.68	11.91 ± 0.75	**<0.001**
Fat mass index	7.37 ± 1.00	5.36 ± 0.58	**<0.001**
Fat mass percentage, %	36.12 ± 3.50	31.02 ± 2.26	**<0.001**
*At 18 months* ^3^			
*n*	12		
Weight-for-age *z*-score	1.92 ± 0.69		
BMI-for-age *z*-score	1.65 ± 0.77		
Length-for-age *z*-score	1.26 ± 0.69		
Waist, cm	48.9 ± 3.3		
Mid upper arm circumference, cm	16.9 ± 1.1		
Head circumference, cm	49.9 ± 1.8		
Lower leg circumference, cm	21.2 ± 1.1		
Thorax circumference, cm	51.4 ± 2.8		
Skinfold, triceps *z*-score	1.81 ± 0.87		
Skinfold, subscapular *z*-score	1.57 ± 0.70		
Bioimpedance, *n*	7		
Fat free mass, kg	8.20 ± 0.58		
Fat mass, kg	4.62 ± 0.75		
Fat free mass index	11.49 ± 0.31		
Fat mass index	6.45 ± 0.72		
Fat mass percentage, %	35.87 ± 2.61		

^1^ Values are expressed as mean ± standard deviation. ^2^ *p*-values for differences between groups by independent *t* test for *z*-scores, and general linear model adjusted for sex for all other variables. HW: High weight-gain group; NW: normal weight-gain group. ^3^ For two infants weight and length at 18 mo was self-reported and there were no other anthropometric data for these two infants.

**Table A2:** Additional blood analysis at 5 and 9 months according to high weight-gain and normal weight-gain group ^1^.

	HW Group	*n* (8–11)	NW Group	*n* (12–14)	*p*-Value ^2^
Albumin, g/L					
5 mo	45.4 [44.7, 46.1]	10	45.9 [44.2, 47.1]	12	0.69
9 mo	46.2 [45.3, 47.7]	8	43.4 [42.7, 45.9]	12	**0.018**
Alkaline phosphatase, U/L					
5 mo	223 [192, 265]	10	239 [210, 278]	12	0.47
9 mo	263 [229, 320]	8	267 [234, 411]	12	0.88
Alanine transaminase, U/L					
5 mo	30 [25, 33]	10	30 [27, 35]	12	0.77
9 mo	26 [22, 32]	8	22 [19, 24]	13	0.095
Aspartate aminotransferase, U/L					
5 mo	52 [43, 64]	10	51 [49, 60]	12	0.84
9 mo	21 [40, 58]	8	43 [41, 49]	13	0.43
Triglyceride, mmol/L					
5 mo	1.37 [1.05, 1.9]	10	1.33 [0.94, 2.08]	12	0.87
9 mo	1.12 [0.98, 2.00]	8	1.40 [1.04, 2.33]	12	0.64
Total cholesterol, mmol/L					
5 mo	3.99 (0.69)	10	3.96 (0.98)	12	0.94
9 mo	3.89 (0.79)	8	3.57 (0.78)	13	0.38
HDL cholesterol, mmol/L					
5 mo	1.01 (0.21)	10	1.03 (0.16)	12	0.81
9 mo	1.01 (0.23)	9	0.94 (0.16)	13	0.40
LDL cholesterol, mmol/L					
5 mo	2.43 (0.57)	10	2.41 (0.97)	12	0.98
9 mo	2.32 (0.66)	9	2.11 (0.79)	13	0.51
CRP, mg/L					
5 mo	0.33 [0.14, 4.88]	10	0.2 [0.10, 055]	12	0.43
9 mo	0.15 [0.06, 0.22]	8	0.24 [0.13, 0.89]	13	0.16
Lactate dehydrogenase, U/L					
5 mo	299.75 [265.3, 309.6]	10	299.15 [268.25, 327.5]	12	0.79
9 mo	291.45 [269.7, 412.25]	8	270.7 [264.55, 300.15]	12	0.25
Bilirubin, µmol/L					
5 mo	5.44 (1.33)	10	5.13 (1.61)	12	0.62
9 mo	4.49 (1.33)	9	3.86 (1.48)	13	0.31
Urea, mmol/L					
5 mo	1.67 [1.40, 1.90]	10	1.77 [1.68, 2.22]	12	0.21
9 mo	2.09 [1.99, 2.37]	8	3.04 [2.38, 3.47]	12	**0.025**
Haemoglobin, mmol/L					
5 mo	7.31 (0.66)	11	7.21 (0.43)	13	0.67
9 mo	7.38 (0.54)	10	6.98 (0.48)	14	0.078
Ferritin, µg/L					
5 mo	33.6 [16.5, 61,5]	10	57.7 [29.7, 68.2]	12	0.26
9 mo	16.7 [11.9, 28,6]	9	24.7 [15.1, 33.2]	13	0.24

^1^ Values are expressed as mean ± standard deviation or median [25th; 75th percentile] as appropriate. HW: High weight-gain group; NW: normal weight-gain group. ^2^ *p*-values for differences between groups by independent *t* test and Mann-Whitney U test as appropriate.
